# Reassessment of *Aeromonas oralensis*. Comment on Mashzhan et al. Whole-Genome Sequencing of a Potentially Novel *Aeromonas* Species Isolated from Diseased Siberian Sturgeon (*Acipenser baerii*) Using Oxford Nanopore Sequencing. *Microorganisms* 2025, *13*, 1680

**DOI:** 10.3390/microorganisms14051080

**Published:** 2026-05-11

**Authors:** Antonio Martínez-Murcia, Aaron Navarro, Caridad Miró-Pina

**Affiliations:** 1 Area of Microbiology, University Miguel Hernández, 03312 Orihuela, Alicante, Spain; 2 genetic PCR solutions^®^, 03300 Orihuela, Alicante, Spain

In this comment, we re-evaluate the proposal of *Aeromonas oralensis* as a novel species by Mashzhan et al. (2025) [1] using phylogenetic and genome-based analyses. The analysis indicated that strain AB005 falls within the diversity of *Aeromonas hydrophila* and does not support its recognition as a distinct species.

*Aeromonas* species are generally recognized pathogens of concern in aquaculture, also able to cause disease in humans [2,3]. *Aeromonas oralensis* sp. nov. was described on the basis of a single strain, AB005, isolated from the ulcerated muscle and gill lesions of diseased Siberian sturgeon (*Acipenser baerii*) at a farm in Oral, Western Kazakhstan [1]. Strain AB005 was first identified as *A. hydrophila* based on 100.0% 16S rRNA sequence identity (1403 bp) to that of the type strain *A. hydrophila* ATCC 7966^T^. However, the 16S rRNA gene has limited resolving power for species delineation within the genus *Aeromonas*, and closely related species often share nearly identical sequences [4]. Despite this high sequence similarity, this isolate did not produce indole and tested negative for ornithine decarboxylase and D-xylose fermentation, which were considered significant differences from typical *A. hydrophila*. The genome of strain AB005 was sequenced and showed an average nucleotide identity (ANI) value of 96.38% with *A. hydrophila* ATCC 7966^T^, 93.24% with its closest species *A. dhakensis* (formerly *A. aquariorum*), and 86.02% with *A. jandaei*. Phylogenomic analyses using Type (Strain) Genome Server (https://tygs.dsmz.de/, accessed on 30 March 2026), Genome-to-Genome Distance Calculator 3.0 (https://ggdc.dsmz.de/, accessed on 30 March 2026), and Ortho Average Nucleotide Identity (OrthoANI Tool v0.93.1) showed a digital DNA–DNA hybridization (dDDH) value of 68.9% with *A. hydrophila* ATCC 7966^T^ and 68.0% with *A. hydrophila* subsp. *ranae* CIO 107985. Although very close to the 70% threshold, the authors considered these values to indicate that the strain AB005 represents a distinct species. Comparative genomic analysis using OrthoVenn v3 revealed several unique gene clusters which, together with the differences observed in the accessory genomes, support the genomic divergence of strain AB005. However, the BLASTp analysis of the unique gene clusters revealed that the highest sequence identities (>95%) corresponded to proteins from *A. veronii* and A. *hydrophila*. Despite sharing significant similarities with *A. hydrophila*, the unique combination of its genomic features, resistance gene profile, and physiological characteristics were significant enough to consider strain AB005 as a novel species.

The identification of aeromonads using biochemical tests often fails due to the high phenotypic variability for strains of the same species, as is widely reported [5]. Currently, phylogenetic analysis and genome-based metrics are the standard framework for reliable species identification. Therefore, we investigated strain AB005 using multilocus phylogenetic analysis (MLPA) of six concatenated housekeeping gene sequences, including the type and other reference strains when available, an approach recommended for *Aeromonas* species delineation [6,7]. As housekeeping genes evolve in concert, the concatenated MLPA phylogeny reflects whole-genome relationships [7]. In addition to the type strain, we included publicly available genomes of *A. hydrophila* that have been previously identified as members of this species [8] for MLPA and genome-based comparisons, allowing for a more comprehensive representation of the genomic diversity of the species. The MLPA was consistent with phylogenomic relationships, and therefore provides a robust phylogenetic frame capable of discriminating *Aeromonas* species [7,9]. As illustrated in Figure 1, the strain AB005 was clearly included within the cluster corresponding to *A. hydrophila*, a robust phylogenetic group with a 100% bootstrap value. In addition, comparison of the 16S rRNA gene copies of strain AB005 with those of the type strain of *A. hydrophila* and other *A. hydrophila* strains included in the phylogenetic analysis revealed sequence similarities of 99.74–100%. These minor differences are attributable to microheterogeneities among the multiple copies of the 16S rRNA gene, a phenomenon previously reported in *Aeromonas* [10]. Moreover, ANI values were calculated using Skani (https://github.com/bluenote-1577/skani, accessed on 30 March 2026) [11]. Strain AB005 showed an ANI value of 96.19% with the type strain of *A. hydrophila* CECT 839^T^ and ANI values ranging from 96.33 to 97.68% with other *A. hydrophila* strains included in the MLPA tree (Figure 1). According to MLPA, *A. dhakensis* and *A. enteropelogenes* were the closest species, with ANI values of 93.89% and 87.93%, respectively, between strain AB005 and the corresponding type strains. More distantly related species such as *A. caviae* and *A. jandaei* showed ANI values of 89.25% and 88.49%, respectively, between strain AB005 and the corresponding type strains (Table 1). These ANI values, which fully agreed with those previously described [1], clearly meet the threshold to consider strain AB005 as belonging to *A. hydrophila*. In addition, we used GGDC [12] to calculate the dDDH of strain AB005 with respect to reference genomes and results were as follows: 68.90% with *A. hydrophila* CECT 839^T^, 68.90–78.10% with the other *A. hydrophila* strains in the MLPA-tree, 49.60% with *A. dhakensis* CECT 5744^T^, 32.30% with *A. caviae* CECT 838^T^, 31.00% with *A. jandaei* CECT 4228^T^, and 29.90% with *A. enteropelogenes* CECT 4487^T^ (Table 1). These values were consistent with those previously reported [1]. Although the dDDH value of 68.90% between strain AB005 and the *A. hydrophila* type strain is slightly below the conventional 70% threshold for species delineation, comparisons with additional *A. hydrophila* strains yield values above this limit, reaching up to 78.10%, supporting the inclusion of strain AB005 within the genomic diversity of the species. Similar cases have been reported in other bacterial taxa, where strains within the same species may exhibit dDDH values below the conventional 70% threshold, while ANI values and phylogenomic analyses consistently support their classification within a single species [13]. Taken together, the present analyses, including MLPA, ANI, and dDDH, unequivocally indicate that the isolate AB005 belongs to *A. hydrophila*, the type species of the genus *Aeromonas*. The atypical phenotypical characteristics reported are not of sufficient taxonomic value to consider a novel taxon; however, they argue for vast diversity within the species *A. hydrophila*. Furthermore, because *A. oralensis* was described from a single isolate and this strain exhibits very high phylogenetic and genomic relatedness to *A. hydrophila*, it was difficult to establish reliable species boundaries. Under such circumstances, the recognition of a separate species does not appear to be justified. According to Rule 24b of the International Code of Nomenclature of Prokaryotes [14,15], in case of heterotypic synonymy of same rank taxa, the senior name, because it was first validated, has priority over the junior. This rule only applies to names that have been validly published [16]. Since *A. oralensis* has not been validly published (https://lpsn.dsmz.de/genus/aeromonas, accessed on 30 March 2026), it has no standing in the nomenclature and cannot be formally proposed as a heterotypic synonym of *A. hydrophila*. Nevertheless, the phylogenetic and genomic evidence presented here clearly indicates that strain AB005, although it may test negative for indole production, ornithine decarboxylase, and D-xylose fermentation, belongs to *A. hydrophila* and does not support its recognition as a distinct species.

## Figures and Tables

**Figure 1:**
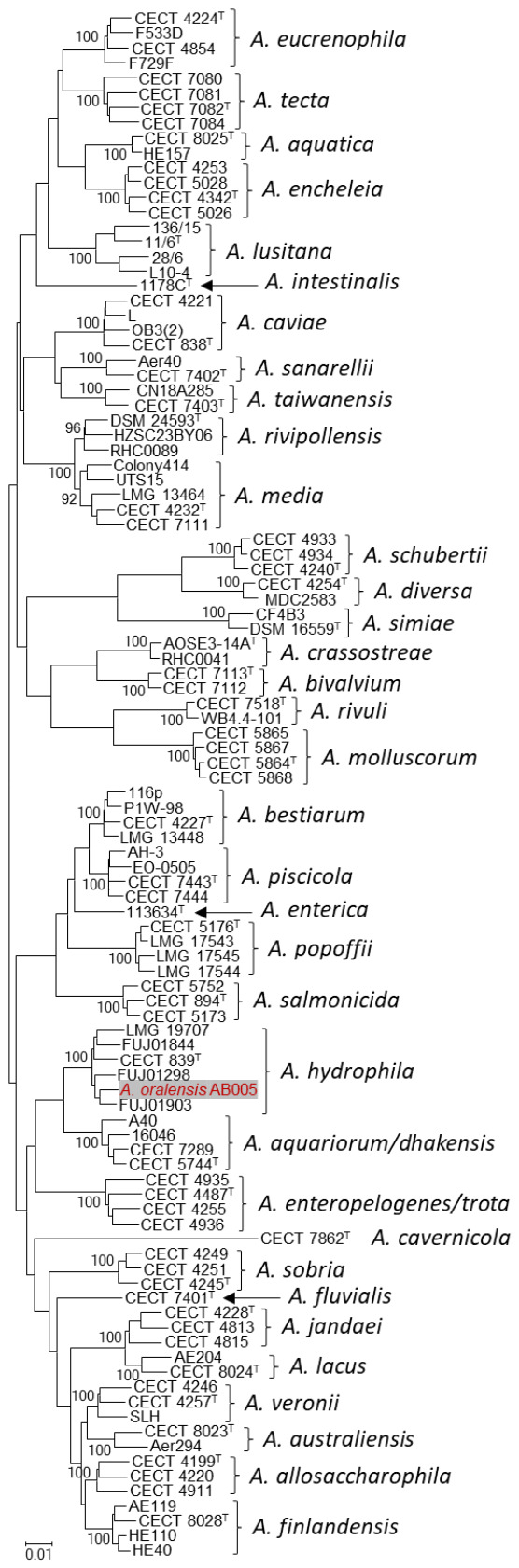
Multilocus phylogenetic analysis (MLPA) obtained from the concatenated sequences of housekeeping genes *gyrB*, *rpoD*, *recA*, *dnaJ*, *gyrA*, and *dnaX* (N-J method; total of 3084 bp) showing the relationships of *Aeromonas* species and strain AB005 (highlighted in red). Numbers at nodes indicate bootstrap values (percentage of 1000 replicates). Bar, 0.01 estimated nucleotide substitutions per site. All sequence data were downloaded from the NCBI website.

**Table 1:** Genome relatedness indices between different *Aeromonas* strains based on average nucleotide identity (ANI) and digital DNA–DNA hybridization (dDDH).

Reference	Query	ANI	dDDH
*A. hydrophila* CECT 839^T^	*A. oralensis* AB005	96.19%	68.90%
*A. hydrophila* FUJ01298	*A. oralensis* AB005	96.33%	68.90%
*A. hydrophila* FUJ01844	*A. oralensis* AB005	96.46%	70.00%
*A. hydrophila* FUJ01903	*A. oralensis* AB005	97.68%	78.10%
*A. hydrophila* CECT 839^T^	*A. hydrophila* FUJ01298	97.15%	74.70%
*A. hydrophila* CECT 839^T^	*A. hydrophila* FUJ01844	97.01%	72.90%
*A. hydrophila* CECT 839^T^	*A. hydrophila* FUJ01903	96.46%	69.20%
*A. hydrophila* CECT 839^T^	*A. dhakensis* CECT 5744^T^	94.04%	50.30%
*A. dhakensis* CECT 5744^T^	*A. oralensis* AB005	93.89%	49.60%
*A. caviae* CECT 838^T^	*A. oralensis* AB005	89.25%	32.30%
*A. jandaei* CECT 4228^T^	*A. oralensis* AB005	88.49%	31.00%
*A. enteropelogenes* CECT 4487^T^	*A. oralensis* AB005	87.93%	29.90%

## Data Availability

The original contributions presented in this study are included in the article. Further inquiries can be directed to the corresponding author.
